# Supplementary material for: High epilepsy prevalence and excess mortality in onchocerciasis-endemic counties of South Sudan: A call for integrated interventions
Source: PLoS Negl Trop Dis. 2025 Jun 30;19(6):e0013244. doi: 10.1371/journal.pntd.0013244 (PMC12237275; doi:10.1371/journal.pntd.0013244)
Supplement: S1 Appendix — (DOCX) [file pntd.0013244.s002.docx]

Supplementary Information

**High epilepsy prevalence and excess mortality in onchocerciasis-endemic counties of South Sudan: A call for integrated interventions**

**Luís-Jorge Amaral^1^, Stephen Raimon Jada^2^, Jane Y. Carter^3^,** **Yak Yak Bol^4^, María-Gloria Basáñez^5^, Charles R. Newton^6,7^, Joseph N Siewe Fodjo^1^, Robert Colebunders^1^**

^1^ Global Health Institute, University of Antwerp, Antwerp, Belgium ([luisjtmamaral@gmail.com](mailto:luisjtmamaral@gmail.com), [JosephNelson.SieweFodjo@uantwerpen.be](mailto:JosephNelson.SieweFodjo@uantwerpen.be), [robert.colebunders@uantwerpen.be](mailto:robert.colebunders@uantwerpen.be)).

^2^ Amref Health Africa, Juba, South Sudan ([stephen.Jada@amref.org](mailto:stephen.Jada@amref.org)).

^3^ Amref Health Africa Headquarters, Nairobi, Kenya ([jane.Carter@amref.org](mailto:jane.Carter@amref.org)).

^4^ Neglected Tropical Diseases Unit, Ministry of Health, Juba, South Sudan ([yakdit16@gmail.com](mailto:yakdit16@gmail.com)).

^5^ UK Medical Research Council Centre for Global Infectious Disease Analysis, and London Centre for Neglected Tropical Disease Research, Department of Infectious Disease Epidemiology, School of Public Health, Imperial College London, London, United Kingdom ([m.basanez@imperial.ac.uk](mailto:m.basanez@imperial.ac.uk)).

^6^ Department of Psychiatry, University of Oxford, Oxford, United Kingdom ([charles.newton@psych.ox.ac.uk](mailto:charles.newton@psych.ox.ac.uk)).

^7^ Neurosciences Unit, Clinical Department, KEMRI-Wellcome Trust Research Programme-Coast, Kilifi, Kenya ([cnewton@kemri-wellcome.org](mailto:cnewton@kemri-wellcome.org)).

**Contents**

[QUESTIONNAIRE A. Maridi 2022 household screening questionnaire used to detect persons with suspected epilepsy. 3](#_Toc199180048)

[QUESTIONNAIRE B. Wulu 2024 household screening questionnaire used to detect persons with suspected epilepsy. 4](#_Toc199180049)

[QUESTIONNAIRE C. Mvolo 2022 household screening questionnaire used to detect persons with suspected epilepsy. 6](#_Toc199180050)

[QUESTIONNAIRE D. Mundri 2021 household screening questionnaire used to detect persons with suspected epilepsy. 7](#_Toc199180051)

[QUESTIONNAIRE E. Clinical, including neurologic questionnaire used by trained clinicians to confirm or exclude epilepsy diagnosis. 8](#_Toc199180052)

[TEXT A. Statistical comparisons and effect sizes of sociodemographic variables across sites. 14](#_Toc199180053)

[TABLE A. Sociodemographic characteristics of the households surveyed per county and classification of study villages, South Sudan. 15](#_Toc199180054)

[TABLE B. Sociodemographic characteristics of the individuals surveyed per county and classification of study villages, South Sudan. 17](#_Toc199180055)

[TABLE C. Anti-Ov16 seropositivity and seroprevalence in 3-9-year-olds per county and classification of study villages, South Sudan. 18](#_Toc199180056)

[TABLE D. Suspected epilepsy prevalence per study county and classification of study villages, South Sudan. 20](#_Toc199180057)

[TABLE E. Positive predictive values (PPVs) of the epilepsy screening question (“whether he/she had been known to have epilepsy or experienced two or more seizures”) per study counties and classification of study villages, South Sudan. 21](#_Toc199180058)

[TEXT B. General interpretation of arcsin-transformed weighted linear regressions 23](#_Toc199180059)

## QUESTIONNAIRE A. Maridi 2022 household screening questionnaire used to detect persons with suspected epilepsy.

## QUESTIONNAIRE B. Wulu 2024 household screening questionnaire used to detect persons with suspected epilepsy.


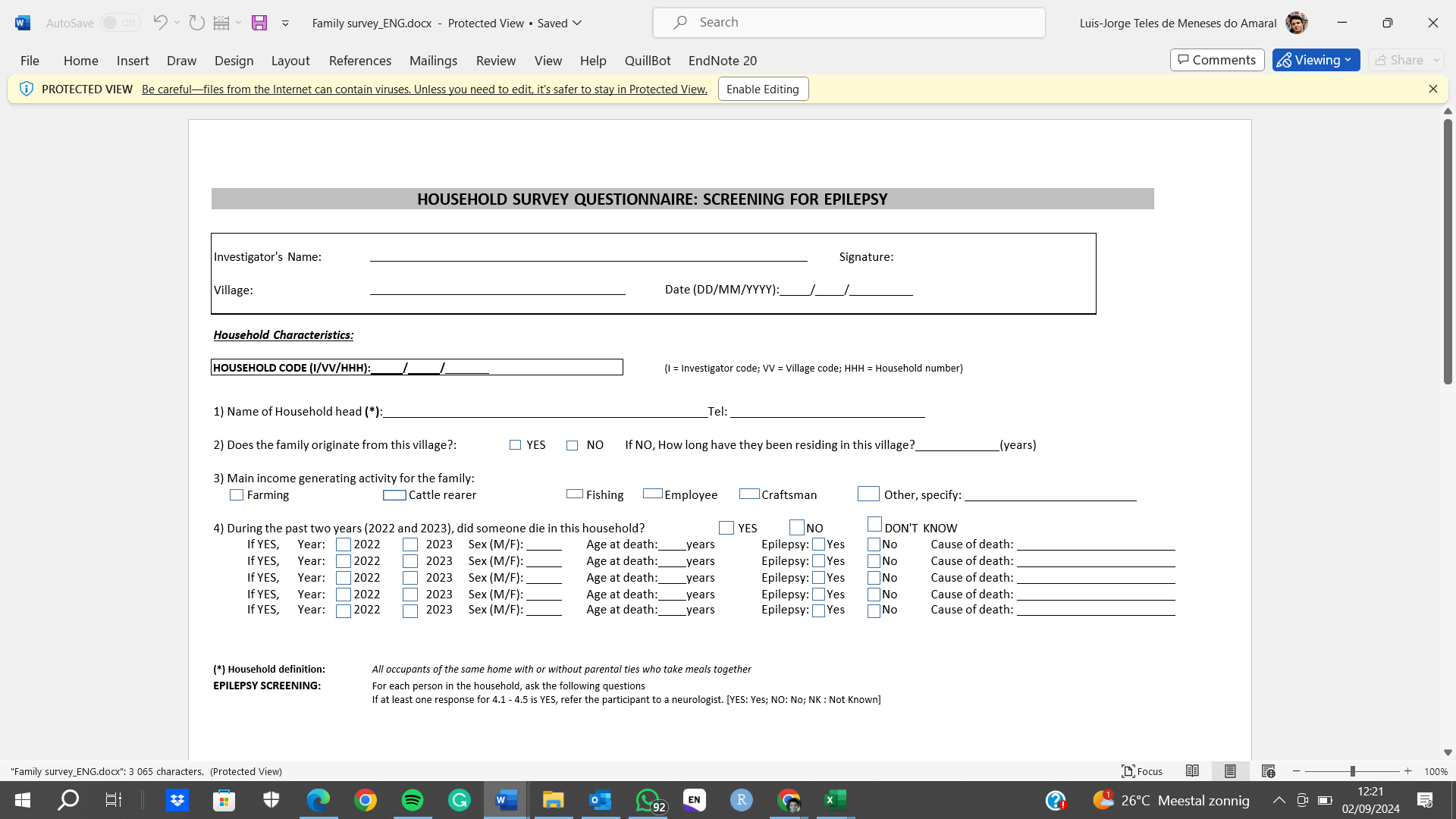


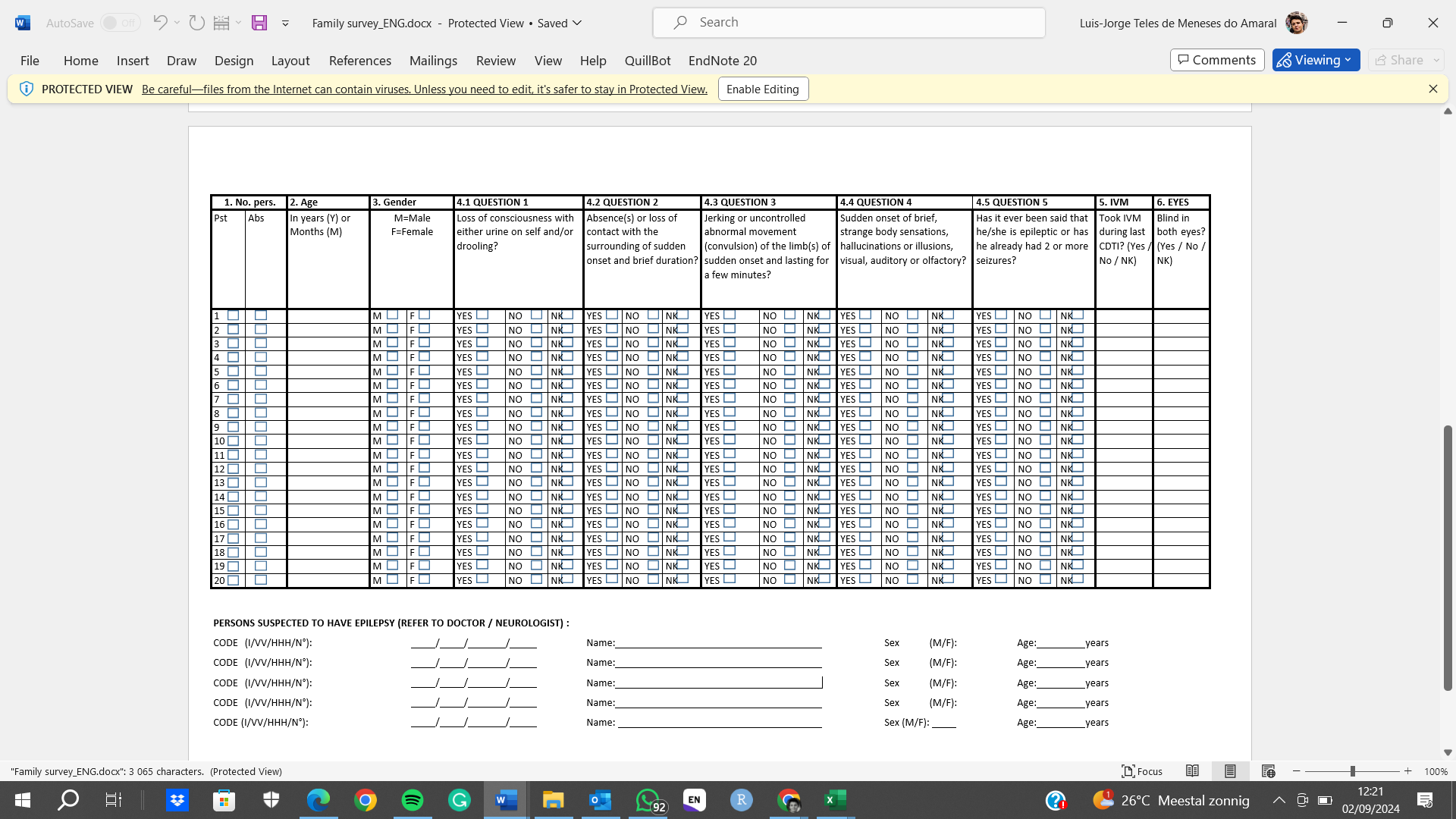


##
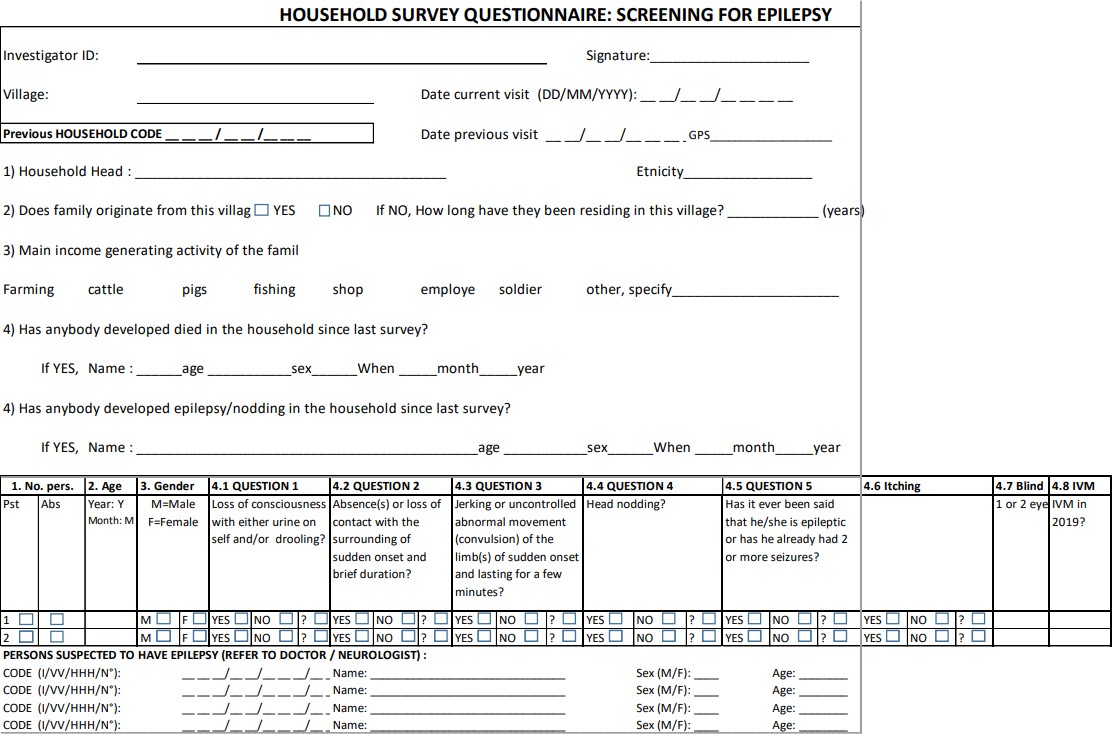
QUESTIONNAIRE C. Mvolo 2022 household screening questionnaire used to detect persons with suspected epilepsy.

*Ethnicity was only asked in the first survey.

## QUESTIONNAIRE D. Mundri 2021 household screening questionnaire used to detect persons with suspected epilepsy.

## QUESTIONNAIRE E. Clinical, including neurologic questionnaire used by trained clinicians to confirm or exclude epilepsy diagnosis.

DATE : _____ / _____ / ________

**PARTICIPANT IDENTIFICATION**

1. Participant ID: Number AS (3 capital letters) number village (2 capital letters) number household (3 digits) number family member (2 digits)
2. First name : __________________________________________________
3. Surname name:_______________________________________________
4. Age years (if less 1year put 0)
5. If less than 1 year …………….month
6. Gender  male  Female
7. Ethnic group:_______________________________________________
8. Was the person living in the village since birth YES NO DON’T KNOW
9. If not, since how long does the person live in the village

________ years (if less than 1 year put 0)

1. What is the interviewed participant’s duration of stay in the survey area __________years

**HISTORY OF EPILEPSY**

***SEIZURE TYPOLOGY***

1. Has the participant ever suddenly lost consciousness YES NO DON’T KNOW

If yes, did s/he experience any of the following?

- - 1. Loss of bladder control? YES NO DON’T KNOW
    2. Foam at the mouth? YES NO DON’T KNOW
    3. Biting of the tongue? YES NO DON’T KNOW

1. Has the participant ever experienced absence(s) or sudden loss(es) of contact with the surroundings, for a short duration of time?

YES NO DON’T KNOW

1. Does the participant have a history of head nodding?  YES, in the past  YES, still ongoing  NO  DON’T KNOW

IF YES, specify age of the participant at onset of head nodding ______ years (if less than 1 year put 0)

1. Has the participant ever experienced sudden, uncontrollable twitching or shaking of arms, legs or head, for a period of a few minutes with amnesia (deficit of memory)?

YES NO DON’T KNOW

1. Does the participant sometimes experience sudden and brief bodily sensations, see or hear things that are not there, or smell strange odours? YES NO DON’T KNOW
2. Has the participant ever been told that he / she is suffering from epilepsy or that he / she has had epileptic fits? YES NO DON’T KNOW

If so, was the diagnosis confirmed by a medical doctor? YES NO DON’T KNOW?

1. Of what type are the most frequent seizures?

Generalized tonic-clonic seizures
  Generalized myoclonic seizures
  Atonic seizures (drop attacks)
  Absences

Nodding seizures
  Focal motoric seizures without loss of consciousness
  Focal motoric seizures with decreased consciousness
  Secondarily generalized partial seizures

Focal non-motor seizures (e.g., hallucinations)

One seizure
  Others, specify:_____________________________

No seizure. If no, skip to other diagnosis (Qn66)

1. At what age the seizures started? ____ year  DON’T KNOW (999)  NA (Not applicable)(888)
2. Did the seizures start less than one year ago?  YES  NO  DON’T KNOW
3. If yes, since how many months? __________________Months
4. What triggers the seizures / head nodding? (Tick all that apply)

Spontaneous (no obvious trigger)
 Sight of food Cold weather

Nothing DON’T KNOW

Other, specify_______________________

***SEIZURE HISTORY***

1. What is the number of epileptic seizures since onset?  One  Two  Three or more seizures

If only two seizures, were they more than 24h apart?  YES  NO  DON’T KNOW  NA

1. Has the participant had a seizure in the last 5 years?  YES  NO  DON’T KNOW
2. Has the participant had a seizure in the last 12 month?  YES  NO  DON’T KNOW
3. What is the current frequency of the seizures?

yearly (if less than 1 per month)

monthly (if less than 4 per month)

weekly (if less than 7 per week)

daily (if more than 7 per week)

NA

Specify number: _____________per ______________(day, week, month, year)

1. a. How many seizures did you have LAST WEEK?

None  1-4 episodes  More than 4  DON’T KNOW

b. What is the average duration of a seizure episode?

less than a minute  1-5 minutes  more than 5 minutes  DON’T KNOW

***MEDICAL HISTORY***

1. Family history of seizures  YES  NO  DON’T KNOW

IF YES, specify who these are (tick all that apply)

Siblings (brother/sister); No. of affected siblings ______
 Father Mother Grandparent(s)

Other, Specify _________________________________

***Pregnancy and Birth:***

1. Did the pregnancy of the mother of the participant proceed normally?  YES  NO  DON’T KNOW
   If NO, specify: ___________________________________
2. Was the participant born at term (pregnancy had completed 9 months)?  YES  NO  DON’T KNOW
3. Was there a delayed cry at birth?  YES  NO  DON’T KNOW

***Psychomotor Development during Childhood:***

**Prior to onset of seizures**

1. Was the child growing normally prior to the onset of the seizures?  Yes  No  DON’T KNOW

IF NO, at what age did the abnormal growing appear? ______ years

1. Did the child learn to do things like other children of his/her age prior to the onset of the seizures?  Yes  No  DON’T KNOW

IF NO, at what age did the learning difficulty start? ______ year

1. Compared with other children of his/her age, did the child appear in any way mentally backward, dull or slow before the onset of the seizures?

Yes  No  DON’T KNOW

IF YES, at what age did it start? ______ years

***Occurrence of severe disease in the past:***

1. Has the interviewed participant suffered from severe measles preceding the onset of epileptic seizures?  YES  NO  DON’T KNOW

If yes how long before the onset of seizures ……………………Years

1. Has the interviewed participant suffered from a severe form of malaria preceding the onset of epileptic seizures?  YES  NO  DON’T KNOW

If yes how long before the onset of seizures ……………………Years

1. Has the interviewed participant suffered from encephalitis/meningitis preceding the onset of epileptic seizures?  YES  NO  DON’T KNOW

If yes how long before the onset of seizures ……………………Years

1. Has the participant had a head injury with loss of consciousness preceding the onset of epileptic seizures?  YES  NO  DON’T KNOW

If yes how long before the onset of seizures ……………………Years

1. Has the participant had a prolonged posttraumatic coma before the onset of epileptic seizures  YES  NO  DON’T KNOW

If yes how long before the onset of seizures ……………………Years

1. Was the onset of epilepsy following another illness?  YES  NO  DON’T KNOW

If YES, specify the illness:______________________________________

1. **PHYSICAL EXAMINATION Done**  YES  NO if no go to 62
2. Thoracic/spinal abnormalities  YES  NO  DON’T KNOW

IF YES, specify _________________________________________________________

1. Facial abnormalities  YES  NO

IF YES, specify ____________________________________________

1. Does the adolescent (> 16years old) /adult looks like a child?  YES  NO  NA

If yes, external signs of sexual development conform to age:  YES  NO  EXAMINATION DECLINED  NA

If NO, specify:

1. girls:  breast not developed  NA
2. girls and boys:  no pubic hear  NA
3. Cervical Lymph nodes  YES  NO
4. Onchocerciasis nodules  YES  NO
5. Itching  YES  NO
6. Burn lesions  YES  NO
7. If yes how many? ______________________
8. Dermatological lesions  NORMAL  opular/nodular pruritic skin  itching without skin lesions  leopard skin  dry, thickened, wrinkled skin  burns  other skin abnormality

Another dermatological lesion specify ________________________

***NEUROLOGICAL EXAMINATION***

1. Is the participant alert?  YES  NO
2. Fully oriented in place/time/person  YES  NO
3. Is the participants mentally impaired?  YES  NO
4. Ophthalmology  NORMAL  ABNORMAL VISION
     BLIND, one eye affected  BLIND, both eyes affected
5. Generalised muscle wasting  YES  NO
6. Paresis  YES  NO
7. Is the participant walking normally?  YES  NO  DON’T KNOW
8. If no specify_____________________________________

***Psychiatric symptoms***

1. Does to participant suffer from another neuro-psychiatric / psychological problem?  abnormal aggressive behaviour  depression  other

IF other, specify: _____________________________________________________________

1. **Physical / Functional Indices**

*Modified Rankin Scale: Please mark the most accurate description of the current functional state of the person with epilepsy, as observed during the evaluation*

| 1 | No significant disability despite symptoms; able to carry out all usual duties and activities |
| --- | --- |
| 2 | Slight disability: unable to carry out all previous activities, but able to look after own affairs without assistance |
| 3 | Moderate disability: requiring some help, but able to walk without assistance |
| 4 | Moderately severe disability: unable to walk without assistance and unable to attend to own bodily needs without assistance |
| 5 | Severe disability; bedridden, incontinent and requiring constant nursing care and attention |

1. ***Epilepsy CASE CLASSIFICATION***  Epilepsy
     Head nodding syndrome

Head nodding syndrome plus
  Other diagnosis

1. If NO epilepsy: other diagnosis?  One seizure

Recurrent febrile convulsions

Dizziness / syncope

Paroxysmal vertigo

Alcohol/drug use

Severe anaemia

Severe protein malnutrition

Psychogenic non-epileptic seizure (PNES)

Mental retardation without epilepsy
  Psychiatric illness without epilepsy

Classic migraine

Other, specify______________________

***ANTI-EPILEPTIC TREATMENT***

1. What is or was the type of seizure medication taken by the participant?

No treatment  DON’T KNOW
  Traditional  anti-epileptic drug
  Mixed  NA

If No treatment or Traditional or Don’t know or NA: Go to Ivermectin use (68)

1. *If anti-epileptic drug treatment or Mixed:* Which substance is taken by the participant?

Phenobarbital
 Sodium valproate
 Phenytoin
 Carbamazepine
 Another anti-epileptic

If other, specify: _____________________________________________

1. Is the participant taking the treatment?

currently every day  currently but with interruptions  only in the past  DON’T KNOW

If interruptions or only in the past, why?
  (Temporary) non-availability of medication
  Lack of financial means to buy medication
  DON’T KNOW
  Other, specify ______________________________

**IVERMECTIN USE**

1. Has the participant ever received ivermectin?  YES  NO  DON’T KNOW  NA

IF YES: Has the participant taken ivermectin during the last CDTi in 2021  YES  NO  DON’T KNOW

**Full physician name** ________________________________-

## TEXT A. Statistical comparisons and effect sizes of sociodemographic variables across sites.

For comparisons across sites, non-parametric Kruskal-Wallis rank sum tests evaluated differences in continuous variables, a method suited to comparing independent samples without normal distribution, with p-values reported. Epsilon-squared effect sizes were calculated to measure the magnitude of these differences, as follows: <0.005 (negligible), 0.005-0.030 (small), 0.031-0.10 (moderate), and >0.10 (large).^1^ Similarly, for categorical variables, Chi-squared tests of independence were applied, with p-values reported and Cramér’s V effect size as follows: <0.05 (negligible), 0.05-0.010 (weak), 0.11-0.15 (moderate), 0.16-0.25 (strong), and >0.25 (very strong).^2^ Detailed results of these analyses are presented in Tables S1 and S2.

## TABLE A. Sociodemographic characteristics of the households surveyed per county and classification of study villages, South Sudan.

| **Variables** | | **County**  (year of survey) | | | | | | | | **Overall** | **Significant difference between counties** | |
| --- | --- | --- | --- | --- | --- | --- | --- | --- | --- | --- | --- | --- |
|  |  | **Maridi**  (2022) | | **Mundri East**  (2021) | **Mundri West**  (2021) | | **Mvolo**  (2022) | **Wulu**  (2024) | |  | **Effect size** | **p-value** |
|  |  | **High-risk sites** | **Low-risk sites** | **Low-risk sites** | **High-risk sites** | **Low-risk sites** | **High-risk sites** | **High-risk sites** | **Low-risk sites** |  |  |  |
| **Households surveyed**  *N* | | 1,248 | 1,003 | 119 | 108 | 137 | 2,400 | 70 | 144 | 5,229 | - | - |
| **Household size** (individuals)  *median (IQR)* | | 6 (4–8) | 6 (5–9) | 7 (5–9) | 7 (6–8) | 7 (5–9) | 6 (5–8) | 6 (5–9) | 6 (5–8) | 6 (5–8) | Negligible | <0.0001 |
| **From a household native to their current village**  *N (%)* | | 1,032 (82.7) | 863 (86.0) | 108 (90.8) | 80 (74.1) | 111 (81.0) | 1,953 (81.4) | 67 (95.7) | 104 (72.2) | 4,318 (82.6) | weak | <0.0001 |
| **Years of residence of non-native households in their current village**  *median (IQR)* | | 4 (2–9) | 3 (2–7) | 7 (3–11) | 6 (5–7) | 8 (3–11) | 6 (3–11) | 3 (3–9) | 3 (2–4) | 5 (3–9) | Negligible | <0.0001 |
| **Household employment activities**  *N (%)* | **Farm** | 1,102 (88.3) | 835 (83.2) | 98 (82.4) | 88 (81.5) | 119 (86.7) | 2,246 (93.6) | 65 (92.9) | 133 (92.4) | 4,686 (89.6) | Strong | <0.0001 |
|  | **Fishing** | 28 (2.2) | 6 (0.6) | 4 (3.4) | 23 (21.3) | 23 (16.8) | 800 (33.3) | 21 (30.0) | 11 (7.6) | 916 (17.5) | Very Strong | <0.0001 |
|  | **Cattle rearing** | 56 (4.5) | 57 (5.7) | 38 (4.3) | 3 (2.8) | 1 (0.7) | 2,183 (14.2) | 5 (7.1) | 3 (2.1) | 3,125 (9.1) | Strong | <0.0001 |
|  | **Pig rearing** | NA | NA | 8 (4.5) | 5 (4.6) | 6 (4.4) | 21 (0.8) | NA | NA | 40 (1.5) | Strong | <0.0001 |
|  | **Other** | 461 (36.9) | 420 (41.9) | 48 (40.4) | 38 (35.2) | 52 (38.0) | 1,014 (42.3) | 4 (5.7) | 11 (7.6) | 2,048 (39.2) | Moderate | <0.0001 |

Abbreviations: N, number; IQR, interquartile range; NA, not available (not assessed).

## TABLE B. Sociodemographic characteristics of the individuals surveyed per county and classification of study villages, South Sudan.

| **Variables** | **County**  (year of survey) | | | | | | | | **Overall** | **Significant difference between counties** | |
| --- | --- | --- | --- | --- | --- | --- | --- | --- | --- | --- | --- |
|  | **Maridi**  (2022) | | **Mundri East**  (2021) | **Mundri West**  (2021) | | **Mvolo**  (2022) | **Wulu**  (2024) | |  | **Effect size** | **p-value** |
|  | **High-risk sites** | **Low-risk sites** | **Low-risk sites** | **High-risk sites** | **Low-risk sites** | **High-risk sites** | **High-risk sites** | **Low-risk sites** |  |  |  |
| Individuals surveyed  *N* | 7,893 | 6,830 | 878 | 790 | 978 | 15,398 | 483 | 907 | 34,051 | - | - |
| Age (years)^a^  *median (IQR)* | 17  (8–30) | 16  (8–30) | 18  (11–31) | 18  (9–30) | 19  (10–30) | 16  (8–28) | 18  (8–30) | 15  (7–29) | 17  (8–29) | Negligible | <0.0001 |
| Sex (female)  *N (%)* | 4,084 (51.7) | 3,573 (52.3) | 469 (53.4) | 418 (52.9) | 516 (52.8) | 7,805 (50.7) | 260 (53.8) | 483 (53.3) | 17,555 (51.6) | Negligible | 0.16 |
| Ivermectin intake in year prior to each survey^b^  *N (%)* | 4,529 (59.0) | 3,601 (53.8) | 295 (34.2) | 376 (49.2) | 555 (57.8) | 3,629 (24.1) | 269 (59.7) | 644 (73.5) | 13,898 (41.6) | Very strong | <0.0001 |

Abbreviations: IQR, interquartile range; N, number.

^a^ There were 33 missing values in Maridi (20 in high-risk sites and 13 in low-risk sites), five in Mvolo and one in Wulu (low-risk sites).

^b^ There were 12 missing values in Maridi (eight in high-risk sites and four in low-risk sites) and 28 in Wulu (11 in the high-risk site and 17 in low-risk sites), where participants were not available during the door-to-door survey, and no household members could confirm their ivermectin intake. This calculation also excluded the participants already deceased at the time of the respective survey.

## TABLE C. Anti-Ov16 seropositivity and seroprevalence in 3-9-year-olds per county and classification of study villages, South Sudan.

| **County**  (year of survey) | **Classification by distance to vector breeding grounds** | **Positive/Total tested (%)** | | | | **Overall anti-Ov16 seroprevalence**  % (95% CI) | **Overall age- and sex-standardised anti-Ov16 seroprevalence**  % (95% CI) |
| --- | --- | --- | --- | --- | --- | --- | --- |
|  |  | **Male aged 3-6 yrs** | **Female aged 3-6 yrs** | **Male aged 7-9 yrs** | **Female aged 7-9 yrs** |  |  |
| Maridi  (2020 & 2023) | High-risk | 29/68 (42.7) | 27/87 (31.0) | 14/33 (42.4) | 23/51 (45.1) | 38.9 (32.8-45.4) | 38.3 (32.2-44.9) |
|  | Low-risk | 5/50 (10.0) | 5/44 (11.4) | 4/36 (11.1) | 4/23 (17.4) | 11.8 (7.3-18.2) | 11.5 (7.1-17.9) |
|  | Overall | 34/118 (28.8) | 32/131 (24.4) | 18/69 (26.1) | 27/74 (36.5) | 28.3 (24.0-33.1) | 27.9 (23.6-32.7) |
| Mundri East  (2021) | Low-risk | NA | NA | NA | NA | - | - |
| Mundri West (2021) | High-risk | 6/38 (15.8) | 3/20 (15.0) | 5/32 (15.6) | 6/33 (18.2) | 16.3 (10.5-24.2) | 16.1 (10.3-24.1) |
|  | Low-risk | 1/23 (4.4) | 1/19 (5.3) | 1/14 (7.1) | 0/15 (0.0) | 4.2 (1.1-12.7) | 4.4 (1.2-12.8) |
|  | Overall | 7/61 (11.5) | 4/39 (10.3) | 6/46 (13.0) | 6/48 (12.5) | 11.9 (7.8-17.5) | 11.8 (7.8-17.4) |
| Mvolo  (2020) | High-risk | 13/54 (24.1) | 8/43 (18.6) | 11/27 (40.7) | 6/26 (23.1) | 25.3 (18.8-33.2) | 26.7 (20.0-34.6) |
| Wulu (2024) | High-risk | 5/17 (29.4) | 8/18 (44.4) | 3/12 (25.0) | 1/8 (12.5) | 30.9 (19.5-45.0) | 32.2 (20.6-46.2) |
|  | Low-risk | 1/19 (5.3) | 0/24 (0.0) | 0/13 (0.0) | 0/11 (0.0) | 1.5 (0.1-9.1) | 1.7 (0.1-9.4) |
|  | Overall | 6/36 (16.7) | 8/42 (19.1) | 3/25 (12.0) | 1/19 (5.3) | 14.8 (9.2-22.6) | 15.4 (9.7-2.3) |
| Total | | 60/269 (22.3) | 52/255 (20.4) | 38/167 (22.8) | 40/167 (24.0) | 22.1 (19.4-25.1) | 22.3 (19.6-25.2) |

CI, confidence interval; NA, not available (not performed); yrs, years.

## TABLE D. Suspected epilepsy prevalence per study county and classification of study villages, South Sudan.

| **County**  (year of survey) | **Classification by distance to vector breeding grounds** | **Suspected epilepsy** | |
| --- | --- | --- | --- |
|  |  | **Number; Total screened** | **Prevalence**  *(95% CI)* |
| Maridi  (2022) | High-risk sites | 404; 7,682 | 5.3  (4.8–5.8) |
|  | Low-risk sites | 186; 6,703 | 2.8  (2.4–3.2) |
|  | Overall | 590; 14,385 | 4.1  (3.8–4.4) |
| Mundri East  (2021) | Low-risk sites | 30; 862 | 3.5  (2.4–5.0) |
| Mundri West  (2021) | High-risk sites | 37; 765 | 4.8  (3.5–6.7) |
|  | Low-risk sites | 26; 960 | 2.7  (1.8–4.0) |
|  | Overall | 63; 1,725 | 3.7  (2.9–4.7) |
| Mvolo  (2022) | High-risk sites | 669; 15,092 | 4.4  (4.1–4.8) |
| Wulu  (2024) | High-risk sites | 46; 462 | 9.9  (7.5–13.2) |
|  | Low-risk sites | 28; 893 | 3.1  (2.1–4.6) |
|  | Overall | 74; 1,355 | 5.5  (4.3–6.8) |
| Total | | 1,426; 33,419 | 4.3  (4.1–4.5) |

CI, confidence interval; N, number; T, total screened.

## TABLE E. Positive predictive values (PPVs) of the epilepsy screening question (“whether he/she had been known to have epilepsy or experienced two or more seizures”) per study counties and classification of study villages, South Sudan.

| **County**  (year of survey) | **Classification by distance to vector breeding grounds** | **Confirmed epilepsy diagnosis**  *N* | **Rejected epilepsy diagnosis**  *N* | **Positive predictive value**  *% (95% CI)* |
| --- | --- | --- | --- | --- |
| Maridi^a^  (2022) | High-risk | 359 | 1 | 99.7  (98.2–100.0) |
|  | Low-risk | 174 | 1 | 99.4  (96.4–100.0) |
|  | Overall | 533 | 2 | 99.6  (98.5–99.9) |
| Mundri East  (2021) | Low-risk only | 26 | 0 | 100.0  (84.0–100.0) |
| Mundri West  (2021) | High-risk | 28 | 7 | 100.0  (85.0–100.0) |
|  | Low-risk | 14 | 2 | 93.3  (66.0–99.7) |
|  | Overall | 42 | 9 | 97.6  (85.9–99.9) |
| Mvolo^b^  (2022) | High-risk only | 483 | 3 | 99.4  (98.1–99.8) |
| Wulu  (2024) | High-risk | 20 | 12 | 62.5  (43.8–78.4) |
|  | Low-risk | 13 | 8 | 61.9  (38.7–81.1) |
|  | Overall | 33 | 20 | 62.3  (47.9–74.9) |
| Total | | 1,116 | 26 | 97.7  (96.6–98.5) |

CI, confidence interval; N, number.

^a^ Data available from a previous survey in the same area of Maridi recorded a positive predictive value for the epilepsy screening question of 98.1% (612/624).^3^

^b^ Data available from a previous survey in the same area of Mvolo recorded a positive predictive value for the epilepsy screening question of 99.7% (633/635).^4^

## TEXT B. General interpretation of arcsin-transformed weighted linear regressions

Relationship between epilepsy prevalence and anti-Ov16 seroprevalence and between probable nodding syndrome (pNS) prevalence and anti-Ov16 seroprevalence (prevalence values were age- and sex-standardised).

**Transformed model**: The prevalence data for epilepsy and pNS were transformed using the arcsine square root transformation to stabilise variance and normalize the distribution. The regression models in their transformed form are expressed as follows:

- Epilepsy prevalence model: $y=\arcsin\left( \sqrt{\frac{Epilepsy prevalence}{100}} \right)=\beta_{0}+\beta_{1}*x_{1}=0.15134+0.00247*\left( antiOv16 seroprevalence \right)$
- pNS prevalence model: $y=\arcsin\left( \sqrt{\frac{pNS prevalence}{100}} \right)=\beta_{0}+\beta_{1}*x_{1}=0.0740+0.0018*\left( antiOv16 seroprevalence \right)$

**Interpretation on the original scale:** To interpret the relationships on the original prevalence scale (as used for the y-axis in Figure 2), the inverse transformation was applied as follows:

- $Epilepsy prevalence \left( \% \right)=\left[ \sin\left( 0.15134+0.00247*\left( Ov16 seroprevalence \right) \right) \right]^{2}*100$
- $pNS prevalence \left( \% \right)=\left[ \sin\left( 0.0740+0.0018*\left( Ov16 seroprevalence \right) \right) \right]^{2}*100$

Example calculations: For the observed range of anti-Ov16 seroprevalence (1.7% to 38.3%):

- At anti-Ov16 seroprevalence of 1.7%: $Epilepsy prevalence \left( \% \right)=\left[ \sin\left( 0.15134+0.00247*\left( 1.7 \right) \right) \right]^{2}*100=2.4\%$
- At anti-Ov16 seroprevalence of 38.3%: $Epilepsy prevalence \left( \% \right)=\left[ \sin\left( 0.15134+0.00247*\left( 38.3 \right) \right) \right]^{2}*100=5.9\%$

**Marginal Effects (Mathematical Derivation):** The average increase in prevalence (marginal effect) per 1.0% increase in anti-Ov16 seroprevalence is derived using:

$$Margingal Effect= sin(2*{(\beta}_{0}+\beta_{1}*\left( antiOv16 seroprevalence \right)))*\beta_{1}*100$$

The average marginal effect:

- Epilepsy prevalence: 0.10% (range across observed values: 0.08-0.12%)
- pNS prevalence: 0.04% (range across observed values: 0.03-0.05%).

Examples:

For an anti-Ov1-6 seroprevalence of 1.7% (smallest observed anti-Ov16 seroprevalence value):

- $Epilepsy=\sin\left( 2*\left( 0.15134+0.00247*1.7 \right) \right)*0.00247*100=0.076\%$
- $pNS=\sin\left( 2*\left( 0.0740+0.0018*1.7 \right) \right)*0.0018*100=0.028\%$

For an anti-Ov1-6 seroprevalence of 38.3% (largest observed anti-Ov16 seroprevalence value):

- $Epilepsy=\sin\left( 2*\left( 0.15134+0.00247*38.3 \right) \right)*0.00247*100=0.085\%$
- $pNS=\sin\left( 2*\left( 0.0740+0.0018*1.7 \right) \right)*0.0018*100=0.051\%$

**References**

1. Cohen J. Statistical Power Analysis for the Behavioral Sciences. 2nd ed. New York: Routledge; 1988.

2. Akoglu H. User's guide to correlation coefficients. Turk J Emerg Med. 2018;18(3):91-93.

3. Colebunders R, Abd-Elfarag G, Carter JY, Olore PC, Puok K, Menon S, et al. Clinical characteristics of onchocerciasis-associated epilepsy in villages in Maridi County, Republic of South Sudan. Seizure. 2018;62:108-115.

4. Raimon S, Dusabimana A, Abd-Elfarag G, Okaro S, Carter JY, Newton CR, et al. High prevalence of epilepsy in an onchocerciasis-endemic area in Mvolo County, South Sudan: a door-to-door survey. Pathogens. 2021;10(5):599.
