# Supplementary material for: High epilepsy prevalence and excess mortality in onchocerciasis-endemic counties of South Sudan: A call for integrated interventions
Source: PLoS Negl Trop Dis. 2025 Jun 30;19(6):e0013244. doi: 10.1371/journal.pntd.0013244 (PMC12237275; doi:10.1371/journal.pntd.0013244)
Supplement: Attachment — Submitted filename: rev_PLOSNTD.docx [file pntd.0013244.s003.docx]

Thank you for considering me to review this manuscript

In your article you found that epilepsy prevalence was 4%—more than twice the regional average—and people with epilepsy were nearly seven times more likely to die prematurely than those without epilepsy. The highest epilepsy prevalence (up to 7%) was observed in villages near fast-flowing rivers, where blackflies—the insects that transmit onchocerciasis—breed. In these same areas, we found that young children had high levels of antibodies against the parasite that causes onchocerciasis (up to 44%), suggesting ongoing transmission despite control efforts. These findings add to the growing evidence that ongoing onchocerciasis transmission may contribute to epilepsy burden. Our results highlight the urgent need to strengthen onchocerciasis elimination programmes and improve access to antiseizure medication for affected communities to potentially reduce new epilepsy cases and prevent epilepsy related deaths.

Given the relevance of the topic discussed in this article, I would recommend the PLOS NTD to consider the manuscript with Minor Revision. Things to consider;

1. Clearly define key terminologies (e.g Epilepsy and probable nodding syndrome) in the methodology section

2. According to international leagues against epilepsy (ILAE) discourages the use of PWE as people with epilepsy as it shows stigmatization. So it would be of great value to consider use of people with epilepsy rather than a short word (PWE).

3. Paragraph two in the introduction section, the author has stated "The epilepsy treatment gap is large in sub-Saharan Africa (SSA), where about 70% of PWE lack adequate care". I think 70% of people with epilepsy lack anti-seizure medications. It is better the author to be clear
